# Serum Heat Shock Protein 70 Concentration in Relation to Polycystic Ovary Syndrome in a Non-Obese Chinese Population

**DOI:** 10.1371/journal.pone.0067727

**Published:** 2013-06-18

**Authors:** Hui Gao, Jie Meng, Mengjing Xu, Shun Zhang, Bishwajit Ghose, Jun Liu, Ping Yao, Hong Yan, Di Wang, Liegang Liu

**Affiliations:** 1 Department of Nutrition and Food Hygiene, Hubei Key Laboratory of Food Nutrition and Safety, School of Public Health, Tongji Medical College, Huazhong University of Science and Technology, Wuhan, People’s Republic of China; 2 MOE Key Lab of Environment and Health, School of Public Health, Tongji Medical College, Huazhong University of Science and Technology, Wuhan, People’s Republic of China; 3 Xiangyang Food and Drug Administration, Xiangyang, People’s Republic of China; 4 Reproductive medicine center, Tongji Hospital, Tongji Medical College, Huazhong University of Science and Technology, Wuhan, People’s Republic of China; Baylor College of Medicine, United States of America

## Abstract

**Background:**

Polycystic ovary syndrome (PCOS) represents the most common cause of anovulatory infertility and affects 6-15% of women of reproductive age. However, the underlying etiology is still poorly understood. In this study, we attempted to examine the association between circulating heat shock protein 70 (Hsp70) concentrations and PCOS in a non-obese Chinese population.

**Methods and Results:**

Human peripheral blood from 52 patients with PCOS and 57 healthy controls, matched for age and BMI, were analyzed. Women with PCOS were found to have significantly higher fasting insulin (FI) levels, as well as Insulin resistance index (HOMA-IR) (*P* < 0.05). Identically, markers of oxidative stress (malondialdehyde (MDA), 8-Hydroxy-desoxyguanosine (8-OHdG), Nitric oxide (NO)) and inflammation (tumor necrosis factor-alpha (TNF-α), C-reactive protein (CRP)) were markedly increased when compared to controls (*P* < 0.05). Elevated serum Hsp70 was positively correlated with IR, oxidative stress and inflammation in PCOS, even after adjustment for age, BMI and gynecologic inflammation (GI). The receiver-operating characteristic curve (ROC) analysis yielded notably different discriminative value for PCOS, with or without an addition of Hsp70 (areas under the curves were 0.884 (95% CI 0.822-0.946) vs. 0.822 (95% CI 0.744-0.900); *P* for difference = 0.015).

**Conclusions and Significance:**

Increased serum Hsp70 levels are associated with the combination of IR, oxidative stress and low-grade chronic inflammation in PCOS individuals, which provides supportive evidence that Hsp70 plays a key role in the pathogenesis of PCOS. More consequent studies were warranted to confirm the clinical utility of circulating Hsp70, especially in diagnosis and prognosis of PCOS and its long-term health cost.

## Introduction

Polycystic ovary syndrome (PCOS) is one of the most common endocrine disorders encountered in reproductive women with a worldwide incidence of 6-15% [1]. The combination of anovulation and hyperandrogenism signifies the classic form of PCOS, which displays the heterogeneous clinical phenotype manifested by hirsutism, irregular menstrual cycles, polycystic-appearing ovaries, infertility, obesity and insulin resistance (IR) [1,2]. Cumulative evidence has suggested that PCOS is also identified as a significant non-modifiable risk factor associated with type 2 diabetes mellitus (T2DM) [3] and women with PCOS are also proposed to have a more rapid conversion to metabolic syndrome [4] and other long-term cardiometabolic risk factors [5].

Despite its prevalence, the etiology of the condition is still poorly understood. It has become apparent that a frequent feature of women with PCOS is IR accompanied by compensatory hyperinsulinemia [6], in addition, increasing evidence shows that IR plays an important role in the pathogenesis of PCOS [7]. As oxidative stress (OS) impairs glucose uptake in tissue and reduces insulin secretion from pancreatic β-cells, the presence of OS is vital to the mechanism of PCOS [8]. Aside from OS and IR, low-grade chronic inflammation might also involve in PCOS and its associations. Actually it is supported by two large prospective studies demonstrating that markers of low grade chronic inflammation independently predict those at high risk for T2DM in women with PCOS [9,10].

Heat shock proteins (Hsps) are a highly conserved, ubiquitously expressed family of heat shock-responsive proteins that participate in cell function modulation and protein homeostasis [11]. Hsps are expressed at low levels under normal physiological conditions while increase dramatically in response to cellular stress [12]. Among a set of Hsps, heat shock protein 70 (Hsp70) has drawn much attention as the most prominent protein [13], with potent antioxidant, anti-inflammatory, and anti-apoptotic properties [12]. Moreover, Hsp70 is present in the peripheral circulation of healthy individuals, even if Hsps are usually considered to be intracellular proteins with molecular chaperone and cytoprotective functions [14,15]. Elevated serum Hsp70 level has also been regarded to denote ovarian damage [16] and disease severity in transgenic mice against oxidative/ischemic stress [17]. Additionally, increased levels of serum or plasma Hsp70 have been observed in several chronic disorders, i.e. T2DM [18], cardiovascular diseases (CVD) [19] and cancer [20]. Nonetheless, the relationship between circulating Hsp70 and PCOS has not been well established.

Obesity has been shown to incite OS in human and plays a pivotal role in inflammatory processes relevant to cardiovascular risk in women with PCOS [10]. However, approximate 20-50% of the women with PCOS are normal weight or lean [1], and may suffer a different pathophysiology of the disorder from those in obese women [21]. Based on these, the aims of the study were (i) to investigate whether there are differences in serum Hsp70 levels between non-obese women with and without PCOS; (ii) to explore the association of circulating levels of Hsp70 between IR, OS and inflammation in women with PCOS; (iii) to determine whether serum Hsp70 could help improve the predictive value for PCOS.

## Methods

### Study population

This study was carried out in accordance with the Declaration of Helsinki (2008) of the World Medical Association, and was approved by the Ethics Committee of Tongji Medical College Ethics Committee. Patients and controls were only entered into the study after informed written consent had been obtained.

Fifty two Chinese women (age range, 21-38 years; BMI range, < 30 kg/m^2^) who fulfilled the inclusion criteria for PCOS detailed below were enrolled in this study. Briefly, all patients were consecutively recruited from the outpatient of Reproductive Center of Tongji Hospital in Wuhan (Hubei, China) between November 2011 and May 2012. All the women with PCOS had significant clinical symptoms or typical ultrasound abnormalities, such as oligomenorrhea, fertility problem, hirsutism or acne. Patients fulfilling at least 2 of the 3 items in accordance with the consensus criteria defined by the European Society of Human Reproduction and Embryology/American Society for Reproductive Medicine consensus 2003 as following [2]: (1) oligo- or anovulation, (2) clinical and/or biochemical signs of hyperandrogenism, and (3) polycystic ovaries (by ultrasound measurement of ovarian volume and number of antral follicles) were enrolled into the PCOS group.

Controls were randomly selected from healthy subjects matching of age and BMI, on the basis of medical history, clinical examinations, and ultrasound. For these fifty seven healthy women, they visited the Reproductive Center just as part of a group check-up for work and lacked specific health problems. None exhibited clinical or diagnostic evidence of PCOS, nor had received any intervention therapy for PCOS. All controls had regular (21-35 day) menstrual cycles.

For both the PCOS cases and controls, we restricted the study subjects to only individuals who had no combined oral contraceptives, lipid-lowering agents or insulin sensitizer within the latest three months, no early history of diagnosed diabetes, nor any other clinically systemic diseases, acute or chronic inflammatory diseases. Information about age, body weight and other personal data (smoking, alcohol consumption, menstruation and gynecologic inflammation (GI) including cervicitis, pelvic inflammation, urethritis and adnexitis) were collected from self-completed, structured questionnaire at the time of the check-up.

### Laboratory investigations

Blood samples were withdrawn from antecubital vein during the early follicular phase defined as day 3-5 of a menstrual cycle for the control group and a spontaneous bleeding episode for the PCOS group. All subjects were sampled at 7:30-9:30 a.m. after an overnight fast. Fasting serum lipid profile including cholesterol (CHO), triglycerides (TG) and glucose (FG) were analyzed using the diagnostic reagent kits (Applygen Technologies Inc., Beijing, China). Serum total testosterone (TESTO), prolactin (PRL), follicle stimulating hormone (FSH), luteinizing hormone (LH) and estradiol (E_2_) were measured using an automated platform. The respective intra- and inter-assay coefficients of variation were 5.5 and 9.7% for TESTO, 5.6 and 8.5% for PRL, 1.6 and 3.2% for FSH, 2.8 and 4.1% for LH, and 3.4 and 6.2% for E_2_. To evaluate the OS status, the lipid peroxidation product malondialdehyde (MDA), the arginine oxygenated product nitric oxide (NO), and the DNA oxidized product 8-Hydroxy-desoxyguanosine (8-OHdG) in serum were measured. To evaluate the inflammation status, C-reactive protein (CRP) and tumor necrosis factor-alpha (TNF-α) in serum were also detected. In brief, for the commercial assay kits, MDA content was determined by spectrophotometric approach based on thiobarbituric acid reactive substances (TBARS) (Beyotime, Shanghai, China), while NO was certained by measuring stable NO end-products-nitrite and nitrate levels [22] (Biovision Company, USA). Enzyme linked immunosorbent assay (ELISA) kits were used to measure the concentrations of fasting insulin (FI) (R&D systems, USA), CRP (R&D systems, USA), TNF-α (R&D systems, USA), 8-OHdG (Cayman Chemical Company, USA) and Hsp70 (Stressgen, USA). All assays were processed within 2 h after withdrawal and conducted in triplicates. Insulin resistance was estimated by the HOMA-IR according to the following formula: HOMA-IR = FG (mmol/L) × FI (mIU/L)/22.5. Beta cell function (HOMA-β cell) was calculated following the formula: HOMA-β cell = 20× FI (mIU/L)/[FG (mmol/L)-3.5].

### Statistical analysis

Descriptive statistics were calculated for all demographic and clinical characteristics of the study subjects. Comparisons between PCOS cases and controls were performed by two-tailed *t* test (continuous variables, normal distribution) or Mann-Whitney U test (continuous variables, skewed distribution). Spearman correlation coefficients and partial correlation coefficients (adjustment for age, BMI and GI) were used to estimate the interrelationship between Hsp70 and the variables of interest. In linear regression analysis, the data were log transformed when necessary to reduce heterogeneity of variances. Statistical analyses were performed using SPSS for windows software version 12.0 (SPSS Inc, Chicago, IL, USA).

To estimate the discriminative value of serum Hsp70 levels on PCOS, receiver-operating characteristic (ROC) curves were plotted and corresponding areas under the curve (AUC) (AUC, #108)were compared using models with or without serum Hsp70 levels. This part of statistical analysis was performed via Stata 11.0 (Stata Corp., College Station, TX, USA).

Where applicable, significance testing was two-sided at a 0.05 significance level.

## Results

Clinical features, baseline hormonal and metabolic parameters screened both in patients with PCOS and healthy control subjects are presented in Table 1. The patient and control groups were well matched for age and BMI. Serum TESTO, LH, and LH to FSH ratio were significantly higher, and FSH levels were notably lower in the PCOS group. The FI was statistically higher in the PCOS than control women, whereas no difference in FG concentrations was observed between groups. Insulin resistance index (HOMA-IR) rather than insulin secretion index (HOMA-β cell) was distinctively different between the two groups. Likewise, the mean TG concentration but not CHO was visibly higher in the PCOS group. In regard to Hsp70, measuring via a highly sensitive assay, higher level was detected in women with PCOS compared with controls (0.25 (0.22-0.29) ng/ml vs. 0.21 (0.19-0.24) ng/ml, respectively; *P* < 0.001).

**Table 1:** A Comparison of Characteristics between PCOS Cases and Controls.

	PCOS	Control	*P* value
No.	52	57	NS
Age (year)	27.15 (4.16)	28.51 (3.63)	0.072
GI, n (%)	10 (19.23)	6 (10.52)	0.200
BMI (kg/m^2^)	22.50 (20.75-26.00)	22.00 (19.75-25.00)	0.293
CHO (mg/dl)	177.00 (159.75-192.25)	175.50 (155.75-190.25)	0.530
TG (mg/dl)	139.07 (80.24-251.05)	98.48 (77.29-127.67)	0.001
TESTO (ng/dl)	46.50 (33.12-64.75)	31.00 (22.22-39.32)	< 0.001
E_2_ (pg/ml)	59.00 (47.00-71.75)	60.00 (48.50-73.50)	0.889
PRL (ng/dl)	13.50 (9.40-18.25)	12.00 (8.75-18.00)	0.568
FSH (mIU/ml)	5.95 (5.00-7.08)	7.10 (6.30-8.12)	< 0.001
LH (mIU/ml)	6.00 (3.00-8.25)	3.00 (2.00-4.25)	< 0.001
LH/FSH	1.03 (0.60-1,99)	0.52 (0.42-0.66)	< 0.001
FI (μU/ml)	10.01 (8.85-13.25)	8.70 (8.10-10.09)	0.002
FG (mmol/L)	5.25 (4.55-5.88)	5.10 (4.70-5.50)	0.736
HOMA-IR	2.30 (1.82-4.04)	2.07 (1.72-2.48)	0.040
HOMA-β cell	115.84 (87.94-145.98)	123.69 (93.44-174.81)	0.219
Hsp70 (ng/ml)	0.25 (0.22-0.29)	0.21 (0.19-0.24)	< 0.001

Abbreviations: GI, gynecologic inflammation; BMI, body mass index; CHO, cholesterol; TG, triglycerides; TSTO, testosterone; E_2_, estrodiol; PRL, prolactin; FSH, follicle stimulating hormone; LH, luteinizing hormone; FI, fasting insulin; FG, fasting glucose; HOMA-IR, homeostasis model assessment of insulin resistance; HOMA-β cell, homeostasis model assessment of beta cell function; Hsp70, heat shock protein70

Data are presented as number (percentage) for categorical data, mean (standard deviation) for parametrically distributed data or median (interquartile range) for nonparametrically distributed data.

**Figure 1:**
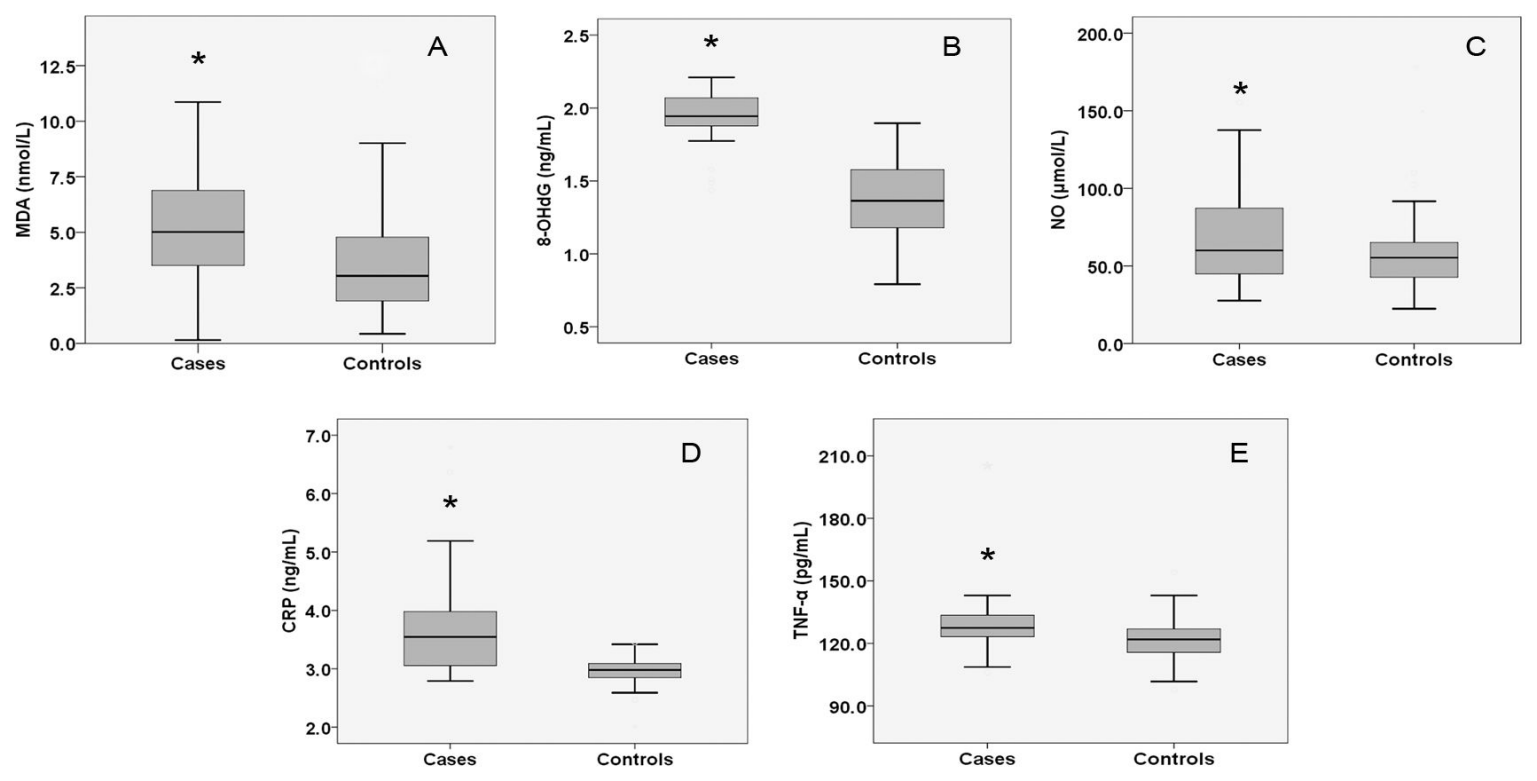
Serum MDA (A), NO (B), 8-OHdG (C), CRP (D) and TNF-α (E) concentrations PCOS individuals and controls. Box plot demonstrating the higher serum levels of MDA (A), 8-OHdG (B), NO (C), CRP (D) and TNF-α (E) in PCOS versus control group (*P* < 0.001, *P* < 0.001, *P* = 0.011, *P* < 0.001 and *P* = 0.001, respectively).

Comparison of the OS and inflammatory biomarkers between the women with PCOS and the controls were conducted via box plot. As shown in Figure 1, the serum MDA (Figure 1A), NO (Figure 1B) and 8-OHdG (Figure 1C) concentrations in PCOS individuals were markedly increased when compared to controls (*P* < 0.05 or *P* < 0.01). Similarly, the inflammatory biomarkers such as CRP (Figure 1D) and TNF-α (Figure 1E) were also considerably higher in PCOS group than control group (*P* < 0.05 or *P* < 0.01).

**Figure 2:**
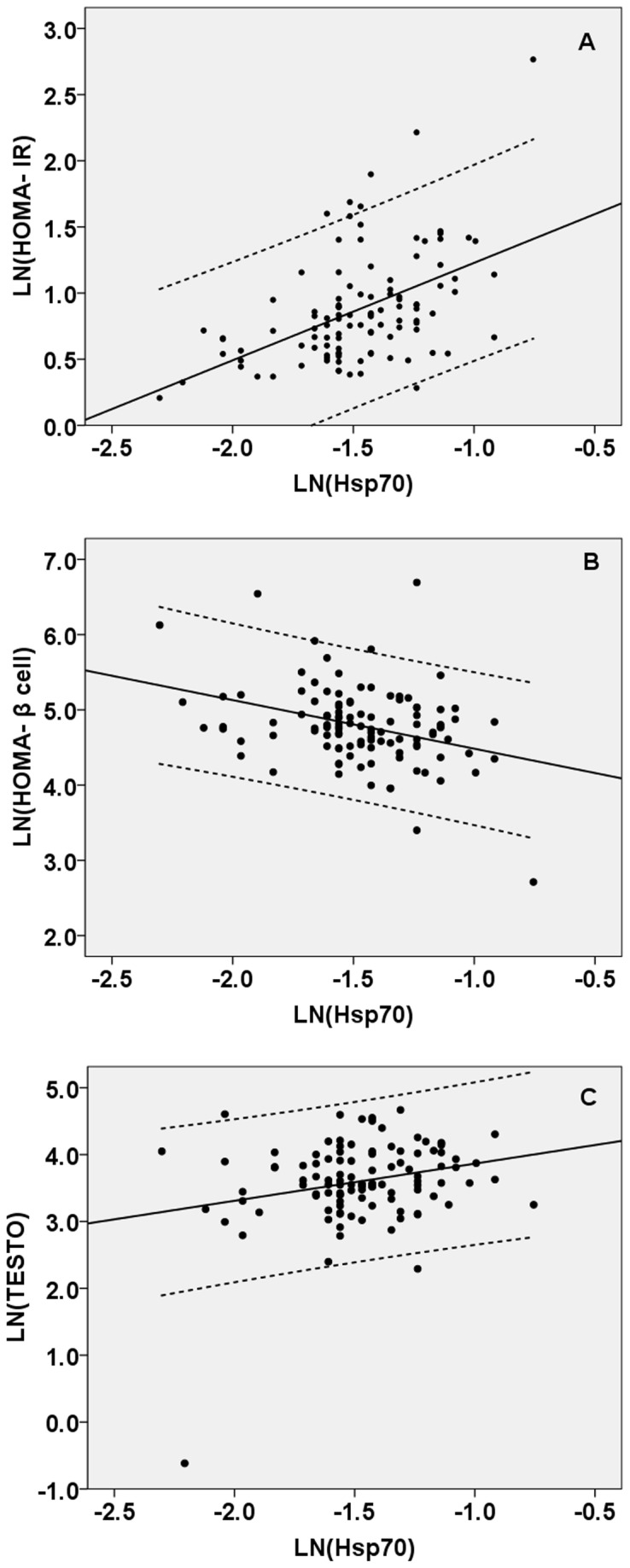
Correlations between the log-transformed serum Hsp70 levels and HOMA-IR (A), HOMA-β cell (B), TESTO (C) for PCOS. Scatter plot demonstrating a significant correlation between the log-transformed serum Hsp 70 levels (ng/ml) and log-transformed HOMA-IR (A) (r = 0.488, *P* < 0.001), HOMA-β cell (B) (r = -0.388, *P* = 0.009) and TESTO (C) (r = 0.250, *P* < 0.001) in patients with PCOS, respectively.

To examine whether there is any relationship between Hsp70 and relevant variables of PCOS, correlation coefficients analysis was performed. What differs is when the interaction comes in. Serum Hsp70 concentrations were significantly positively correlated with BMI, FG, FI, TESTO, MDA, 8-OHdG, NO, CRP and TNF-α concentrations, and HOMA-IR, while negatively correlated with HOMA-β cell and FSH (*P* < 0.05 or *P* < 0.01). In partial correlation analysis, after adjustment for age, BMI and GI, these correlations between serum Hsp70 concentrations and other variables above were attenuated (Table 2). In linear regression models, for each individual, logarithmic transformed HOMA-IR, HOMA-β cell values and TESTO levels was plotted against the logarithmic transformed concentrations of serum Hsp70 concentrations. Obviously, the data showed that serum Hsp70 levels displayed positive dose–response relationships with HOMA-IR values and TESTO levels, whereas negative to HOMA-β cell values, with the linearly dependent coefficients of 0.488 (*P* < 0.001), 0.250 (*P* < 0.001) and -0.388 (*P* = 0.009); respectively (Figure 2).

**Table 2:** Spearman Correlation Coefficients between Serum Hsp70 Levels and Other Variables in the Study Subjects.

Variables	Unadjusted		Adjusted for Age, BMI and GI	
	r	*P* value	r	*P* value
Age	-0.029	0.768	/	/
BMI	0.322	0.001	/	/
GI	0.065	0.502	/	/
CHO	0.181	0.060	0.162	0.111
TG	0.155	0.108	0.034	0.741
FG	0.435	< 0.001	0.247	0.010
FI	0.372	< 0.001	0.258	0.014
HOMA-β cell	-0.288	0.002	-0.174	0.087
HOMA-IR	0.486	< 0.001	0.437	< 0.001
TESTO	0.187	0.050	0.062	0.505
E_2_	-0.094	0.303	-0.036	0.701
PRL	-0.057	0.533	0.098	0.291
FSH	-0.236	0.009	-0.163	0.108
LH	0.079	0.383	0.174	0.087
8-OHdG	0.561	< 0.001	0.553	< 0.001
MDA	0.551	< 0.001	0.447	< 0.001
NO	0.231	0.022	0.163	0.078
CRP	0.683	< 0.001	0.592	< 0.001
TNF-α	0.627	< 0.001	0.542	< 0.001

Abbreviations: 8-OHdG, 8-Hydroxy-desoxyguanosine; MDA, malondialdehyde; NO, nitric oxide; TNF-α, tumor necrosis factor-alpha; CRP, C-reactive protein

The analyses are based on the data from both cases and controls. Spearman correlation coefficients were used to estimate the interrelationship between Hsp70 and the variables of interest. After adjustment for age, BMI and GI, partial correlation coefficients were calculated between Hsp70 and other variables.

**Figure 3:**
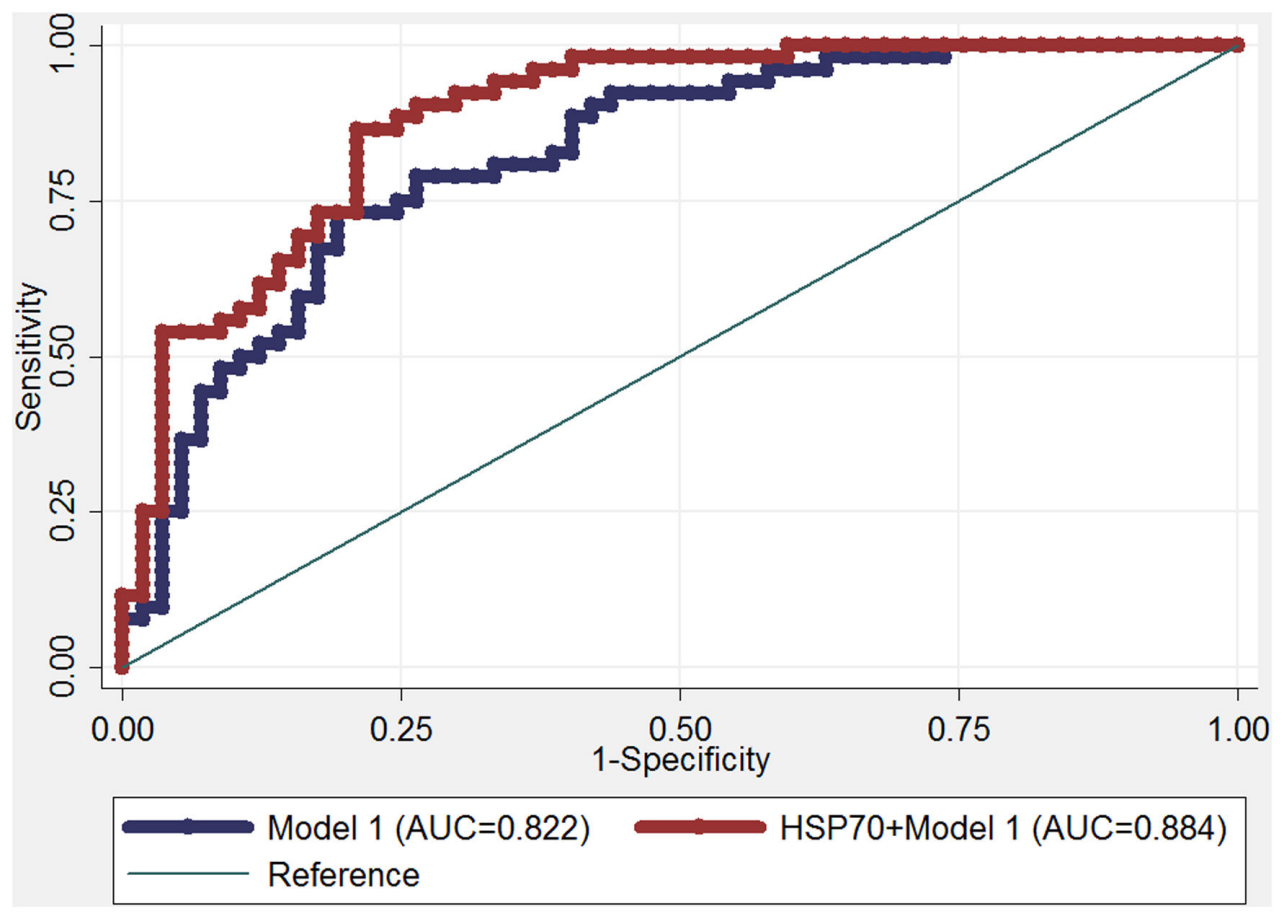
ROC curves and corresponding AUCs for PCOS using models without or with serum Hsp70 level. The AUC in model 1 was 0.822 (95% CI 0.744-0.900), and that in a model with serum Hsp70 level plus the model 1 was 0.884 (95% CI 0.822-0.946); *P* = 0.015 for the difference of the AUCs. The established conventional model here consists of age, BMI, GI, HOMA-IR, CHO and TG.

The ROC curves were performed from the logistic regression models following the recommendation given by Mario [23]. As shown in Figure 3, the AUC for a model with known risk factors (Model 1), comprising age, BMI, GI, HOMA-IR, CHO and TG, was 0.822 (95% CI 0.744-0.900) for PCOS. However, when serum Hsp70 concentration was added in the model (Model 2, including Model 1 plus Hsp70), the AUC was significantly increased to 0.884 (95% CI 0.822-0.946; *P* = 0.015 for the difference of the AUCs).

## Discussion

The increasing worldwide prevalence of PCOS, along with its wide ranging associations, has led to hopes that the disease can be reversed or prevented [1,24]. The current study provides novel findings that serum Hsp70 concentration was elevated in non-obese individuals with PCOS compared to age- and BMI-matched healthy controls, accompanying notable positive correlations with HOMA-IR, increased OS and intrinsic inflammation, which make serum Hsp70 as an emerging independent marker for PCOS.

Increased IR is viewed as a central feature of PCOS and with a prevalence of 25-60% in women with PCOS [6,25]. Both lean and obese women with PCOS have peripheral IR and hyperinsulinemia [26]. The IR associated with obesity or habitual consumption of fatty diets has been linked to increased cellular levels of stored TG and fatty acid derivatives [27,28]. Several studies have implicated that increased expression of Hsp70 is involved in the pathogenesis of insulin-resistant disorders such as the metabolic syndrome [12] and T2DM [18] in the general population. This involvement appears to be bidirectional, because not only does Hsp70 accumulation favor IR and may contribute to pancreatic β-cell dysfunction and diabetes, but also IR may in turn facilitate Hsp70 accumulation within the body [29]. This is in line with the data from our group clarifying that Hsp70 had a positive correlation to HOMA-IR while was negatively correlated with HOMA-β. Analogously, a beneficial effect of Hsp70 on IR has been reported in transgenic animals [30]. Interestingly, expression patterns in our subjects displayed that women with PCOS had higher levels of TG induced by IR, and the elevated Hsp70 intensively correlated with IR, which mirror closely those concepts that IR correlates well with the quantity of TG [31] and there is a good reason to suspect the possibility of a specific link between IR and Hsp70. Similar observations were made recently by Marucci et al. [32] and Nakhjavani et al. [18], who noted that induction of Hsp70 against histological impairment of β-cell was associated with IR, although, in contrast to the present work, Kurucz et al. observed a reduced expression of related Hsp70 gene in Caucasion subjects with T2DM [33], perhaps duo to differences in the ethnicity and gene determinant risk of diabetes.

It is intriguing to note that there is an increase of OS in PCOS and its associations where hyperglycemia and / or hyperinsulinemia take place [34]. Generally, OS occurs when oxidative substances disturb the oxidant-antioxidant balance in human body that causing oxidative damages to nucleic acid, proteins and lipids. As a result, the release of ROS can trigger circulating markers of OS abnormal in women with PCOS and leads to oxidation and aggregation of vital proteins and DNA, ultimately resulting in the failure of normal cell function [11]. Anti-oxidant status of circulating is the result of both anti-oxidant levels (NO) and degree of OS (e.g. MDA and 8-OHdG) [35]. Elevated serum NO, MDA and 8-OHdG in PCOS patients, which separately serve as a marker of lipid peroxidation, protein oxidation and DNA damage in tissues were observed in the present study. Our data extend the finding that OS is associated with the pathogenesis of PCOS [36] and the extent of lipid peroxidation is a fundamental parameter of PCOS [37]. The Hsp70, particularly, confers protection against conditions such as hypoxic / OS [12], thus it is not surprising that serum Hsp70 level was significantly elevated in patients with PCOS. Although the expression of Hsp70 in our study following exposure to stressful conditions in PCOS did not result in link to NO levels, overall, positive correlations with MDA and 8-OHdG further confirms the fact that Hsp70 is in great relationship with OS, as well as lipid peroxidation in PCOS. Our findings are consistent with the results of others that human circulating Hsp70 was increased in response to heat stress [38], and the expression of Hsp70 largely depends on the cellular redox state and OS [39].

There is also evidence of elevations of circulating inflammatory molecules in PCOS [40,41]. In accord with these, marked increase in serum CRP and TNF-α were observed in women with PCOS compared to age and BMI matched controls, which may corroborate existing molecular evidence of the chronic low-grade inflammation underpinning the pathogenesis of this disorder [40]. CRP has emerged as the most promising cardiovascular event risk marker that may amplify the risk of T2DM and death from cardiovascular disorder [42], while TNF-α has served as a known mediator of IR. The combination of elevated CRP and TNF-α in these non-obese young women coalesces sufficiently that PCOS may link to the development of T2DM and cardiovascular disorder. On the other hand, considerable evidence demonstrates that Hsp70 attenuates inflammation via a direct response to inhibit inflammatory mediators or indirectly by suppressing downstream gene transcription of cytokines [43], suggesting a cytoprotective and an anti-autotoxicity roles for intracellular Hsp70 [44,45]. Our findings clearly clarified that serum Hsp70 was positively correlated with high levels of CRP and TNF-α in patients with PCOS. This coincides with the recent notions by Wieten et al. [46] and Cohen [47], who revealed that Hsp70 is up-regulated in inflamed tissue and may constitute a reliable sensor system for the inflammatory state.

Women with PCOS are always accompanied by IR, OS and chronic low-grade inflammation [7,40,48]. Upon hyperglycemia, stimulation of ROS generation from mononuclear cells (MNCs) may serve as an inflammatory trigger for the induction of IR in PCOS [34]. Herein PCOS and its metabolic complications may be explained by the existence of a potential interaction among the three above and the clinical heterogeneity may be explained by the individual variability in the relative contributions of this interaction. Although it has been well known that PCOS is a major risk factor for developing T2DM, CVD and cancer [49–51], however, clinical studies on its biomarkers are limited. As a stress inducible protein, Hsp70 has been accepted as a highly sensitive and reliable biomarker in some chronic disorders, involving CVD [52] and cancer [16]. It seems that the effects of Hsps on reproduction should not be ruled out, like other processes present in the ovary [18,53]. Moreover, the predictive value revealed by ROC curves analysis on Hsp70 level indicates it should be a paramount risk factor and may prove to be of some clinical relevance for PCOS.

It should be acknowledged, however, that our study still has several limitations despite exhibition of a strong association between serum Hsp70 and PCOS. First, case-control study design defeats us to establish temporal relationship between Hsp70 and the prevalence of PCOS, consequently these findings should be confirmed in further prospective cohort studies. In addition, all participants in this study were of Chinese Han ethnicity, which minimizes the confounding effects by ethnic background. Whether these results can be generalized to other populations need further studies.

Taken together, this study provides the evidence that non-obese women with PCOS had higher Hsp70 concentrations than age and BMI matched healthy women. Since IR and OS, as well as chronic low-grade inflammation play vital roles in the pathogenesis of PCOS and its associations, it is likely that a biomarker in reducing the joint action will make a significant impact on the predict of PCOS, such as Hsp70. Whereby that, Hsp70 may serve as a potential biomarker, and may provide an adjunctive method for global assessment of PCOS risk.
